# Potentiality of Self-Cloned *Lactobacillus plantarum* Taj-Apis362 for Enhancing GABA Production in Yogurt under Glucose Induction: Optimization and Its Cardiovascular Effect on Spontaneous Hypertensive Rats

**DOI:** 10.3390/foods9121826

**Published:** 2020-12-09

**Authors:** Farah Salina Hussin, Shyan Yea Chay, Mohammad Zarei, Anis Shobirin Meor Hussin, Wan Zunairah Wan Ibadullah, Nurul Dhania Zaharuddin, Hazrati Wazir, Nazamid Saari

**Affiliations:** 1 Department of Food Science, Faculty of Food Science and Technology, University Putra Malaysia, Serdang Selangor 43400, Malaysia; farahsalina@unikl.edu.my (F.S.H.); shyan_yea@upm.edu.my (S.Y.C.); shobirin@upm.edu.my (A.S.M.H.); wanzunairah@upm.edu.my (W.Z.W.I.); andee25@gmail.com (N.D.Z.); hazratiwazir@gmail.com (H.W.); 2 Section of Food Engineering Technology, Malaysian Institute of Chemical and Bio-Engineering Technology, Universiti Kuala Lumpur, Alor Gajah, Melaka 78000, Malaysia; 3 Department of Food Science and Technology, School of Industrial Technology, Faculty of Applied Sciences, Universiti Teknologi Mara, Shah Alam, Selangor 40450, Malaysia; zarei@uitm.edu.my

**Keywords:** antihypertensive, fermentation, GABA, optimization, rat study, yogurt

## Abstract

The current study evaluated the γ-aminobutyric acid (GABA) producing ability from three novel strains of lactic acid bacteria (*L. plantarum* Taj-Apis362, assigned as UPMC90, UPMC91, and UPMC1065) co-cultured with starter culture in a yogurt. A combination of UPMC90 + UPMC91 with starter culture symbiotically revealed the most prominent GABA-producing effect. Response surface methodology revealed the optimized fermentation conditions at 39.0 °C, 7.25 h, and 11.5 mM glutamate substrate concentration to produce GABA-rich yogurt (29.96 mg/100 g) with desirable pH (3.93) and water-holding capacity (63.06%). At 2% glucose to replace pyridoxal-5-phosphate (PLP), a cofactor typically needed during GABA production, GABA content was further enhanced to 59.00 mg/100 g. In vivo study using this sample revealed a blood pressure-lowering efficacy at 0.1 mg/kg GABA dosage (equivalent to 30 mg/kg GABA-rich yogurt) in spontaneously hypertensive rats. An improved method to produce GABA-rich yogurt has been established, involving shorter fermentation time and lower glutamate concentration than previous work, along with glucose induction that omits the use of costly PLP, fostering the potential of developing a GABA-rich functional dairy product through natural fermentation with desirable product quality and antihypertensive property.

## 1. Introduction

Hypertension is an underlying health complication that relates directly to the prevalence of cardiovascular diseases, including heart failure, coronary artery disease, stroke, atrial fibrillation, and peripheral vascular disease [1]. A continuous hypertension without proper treatment may lead to kidney failure, cognitive decline, and dementia [2]. According to WHO [3], 25% of men and 20% of women were diagnosed with hypertension in the year 2015, resulting in the occurrence of worldwide premature death. Along with the increasing health awareness and consumer demand towards food products that provide beyond basic nutrition (carbohydrate, protein, fat), food scientists are driven towards developing functional foods with additional health benefits.

One of the ingredients receiving such attention is gamma-aminobutyric acid (GABA), a non-proteinaceous amino acid that has been demonstrated to play a vital role in both the central and peripheral nervous system to consequently regulate blood pressure [4,5]. Numerous health benefits have been associated with GABA, including antihypertensive, antidiabetic, antistress, antidepression, and tranquilizing effects for patients suffering from neurological disorders [6]. GABA is naturally produced in various fermented foods, including sourdough [7], cheese [8], fermented milk [9], and fermented sausage [10], mainly as a result of lactic acid bacteria (LAB) metabolism. In LAB, L-glutamate substrate is actively converted into GABA via the irreversible α-decarboxylation reaction, catalyzed by the presence of glutamic acid decarboxylase (GAD) enzyme in the microorganism. However, previous studies revealed that the effective production of GABA required a high concentration of glutamate (20–507 mM), the presence of costly pyridoxal-5-phosphate cofactor (PLP, 18–200 µM), and a long fermentation time (48–120 h) [9,11,12]. These major obstacles make up a tall order that deters the production of GABA-rich fermented foods under minimum usage of glutamate and PLP at a reduced incubation time.

While many studies reported the use of single-strain LAB to generate GABA, only few reported the production of GABA by co-culturing of different bacterial strains. For instance, Watanabe [13] and Kim et al. [14] demonstrated the ability of combined strains, i.e., *S. thermophiles* IFO13957 with *L. bulgaricus* IAM1120 and *L. brevis* GABA 100 with *Bifidobacterium bifidum* BGN4, respectively. In this study, self-cloned and expressed *L. plantarum* Taj-Apis362 recombinant cells, UPMC90 (intracellular) and UPMC91 (extracellular), previously engineered to have high GAD activity by Tajabadi et al. [15], were used to improve the GABA production in yoghurt and can be considered safe since modified organisms, by the self-cloning technique, are now not viewed as genetic modified organisms (GMOs) and are regarded as safe and suitable for food applications [16]. Following this, using yogurt as an example of a fermented food system, the current study performed response surface methodology (RSM) analysis to optimize the fermentation conditions (temperature, glutamate concentration, and incubation time) to maximize GABA production using the co-culturing technique, i.e., a combination of starter culture (*S. thermophilus* and *L. delbrueckii* ssp. *bulgaricus*) and high GABA-producing LAB strain (*L. plantarum* Taj-Apis362), prior to glucose induction to further enhance the GABA content. The final product with suitable pH and water-holding capacity was then administered to spontaneously hypertensive rats (SHR) for in vivo evaluation of blood pressure-lowering efficacy.

## 2. Materials and Methods

### 2.1. Materials

Non-fat skim milk powder (Sunlac^®^ brand) and pasteurized fresh milk (Goodday^®^ brand) were locally purchased. de Man, Rogosa and Sharpe (MRS) broth and MRS agar were obtained from HiMedia Laboratories Pvt. Ltd. (Mumbai, India). HPLC-grade ethanol (99.8%) was purchased from Thermo Fisher Scientific (Waltham, MA, USA) while HPLC-grade acetonitrile was bought from Mallinckrodt Baker, Inc. (J.T. Baker^TM^ brand, Phillipsburg, NJ, USA). GABA standard, glutamate substrate, triethylamine, and phenylisothiocyanate were purchased from Merck KGaA (Darmstadt, Germany). All other chemicals used were of analytical grade.

### 2.2. Preparation of Starter Culture and GABA-Producing LAB Strains

Commercial starter culture, consisting of *S. thermophilus* and *L. delbrueckii* ssp. *bulgaricus* was manufactured by YogurtBio (Lactina^®^ brand, Sofia, Bulgaria). Two self-cloned *L. plantarum* Taj-Apis362 strains possessing high intracellular GAD activity (UPMC90) and high extracellular GAD activity (UPMC91) and a wild-type *L. plantarum* Taj-Apis362 (UPMC1065) were obtained from the culture collection of Institute of Bioscience, Universiti Putra Malaysia (Selangor, Malaysia). The wild-type *L. plantarum* Taj-Apis362 (UPMC1065) was previously isolated from the stomach of honeybee *Apis dorsata*, and used as a host for GAD gene overexpression to produce UPMC90 and UPMC91 strains. Due to the presence of extra GAD-producing gene as a result of genetic material exchange in self-cloned strains, the GAD activity increased by seven-fold compared to the wild-type host [15,17]. Samples were routinely stored in sterile MRS broth at −80 °C as stock culture. All procedures involving the use of *L. plantarum* Taj-Apis strains received approval from National Board of Biosafety, Ministry of Natural Resources and Environment, Malaysia (approval no. JBK [S]-602-1/2/207).

Prior to the production of a new batch of yogurt, starter culture and three LAB strains were freshly prepared before fermentation. Reconstituted skim milk was prepared according to the modified method of Sandoval-Castilla et al. [18]. Briefly, pasteurized fresh milk and skimmed milk powder were mixed to achieve 16% (*w*/*v*) non-fat total solid in the final volume, then subjected to thermal treatment at 80–85 °C for 30 min [19], rapidly cooled to 4 °C in an iced bath, and stored at 4 °C before use. Starter culture was inoculated in the sterilized milk and incubated at 42 °C for 6 h until reaching pH 4.5–4.6. On the other hand, LAB strains were streaked on three different MRS fresh agar plates for single colony isolation, then individually transferred to 10 mL of MRS broth to allow rapid cell growth, incubated at 37 °C for 18 h, and finally sub-cultured at 37 °C for 22–24 h in 10 mL of sterile reconstituted skimmed milk. At the end of the incubation, a thick semi-solid layer of bacterial mass (curd) was formed and used for yogurt production, as detailed in Section 2.3.

### 2.3. Yogurt Fermentation

On the day of fermentation, bacterial curd (starter culture and LAB, having a viable cell count of >10^6^ CFU/g) were mixed at a ratio of 2:1 (weight of starter culture to weight of LAB) into sterilized reconstituted skim milk to produce 5 different samples: control (denoted as S, contained only starter culture), S+UPMC90, S+UPMC91, S+UPMC90+UPMC91, and S+UPMC1065. Glutamate substrate was added at 5 mM and fermentation was allowed at 36 °C for 10 h [15]. For glucose-induced samples, 1–4% glucose (*w*/*v*) was added to four different samples prior to fermentation at the optimized conditions obtained. Glucose was selected based on a preliminary study that evaluated the effect of different simple sugars and prebiotics to enhance GABA in a yogurt system (data not shown). Samples were taken at hourly intervals, rapidly cooled in an iced bath to stop fermentation, and stored in a freezer at −20 °C prior to further analysis.

### 2.4. Experimental Design for RSM Study

Optimization of fermentation conditions was performed using a central composite design to maximize GABA yield. Three independent variables were selected, namely temperature (35–43 °C), glutamate concentration (2–30 mM), and incubation time (4–10 h). The response variables were GABA content, pH, and water-holding capacity (WHC). A total of 20 runs, consisting of different temperatures, glutamate concentrations, and incubation times, were performed in randomized order (Table 1) and in triplicate to minimize the effect of unexplained variability in the actual responses due to extraneous factors. At the end of fermentation, samples were either directly measured for pH or rapidly cooled in an iced bath, then stored at −20 °C for GABA and glutamate determination or stored at 4 °C for WHC analysis.

Regression analysis and analysis of variance (ANOVA) were performed to determine the regression coefficients and statistical significance of the model terms. Model competency was monitored using model *p*-value, lack-of-fit *p*-value, and coefficient of determination (*R*^2^), of which statistically significant terms with *p*-values < 0.05 were included in the reduced model. For linear terms, either significant (*p* < 0.05) or non-significant (*p* > 0.05), were all retained in the final reduced model. Progressively, three-dimensional (3-D) response surface plots were employed to show the relationship between responses and independent variables. All experimental design and data analysis was performed using Design-Expert^®^ software (version 7, Stat-Ease Inc., Minneapolis, MN, USA).

### 2.5. Determination of GABA Content

GABA content was determined following the method previously described by Tajabadi et al. [15] using an HPLC system (Shimadzu LC 20AT, Shimadzu Corporation, Kyoto, Japan) equipped with an oven (model CT0-10ASVP), pump system, and PDA detector (model SPD-M20A). The separation column (endcapped Chromolith^®^ RP-18, 100 mm length × 4.6 mm internal diameter) was supplied by Merck KGaA (Darmstadt, Germany). Briefly, 1.0 g of yogurt sample was centrifuged at 10,000× *g* at 4 °C for 15 min and 10 μL of the supernatant were collected in small durham tube for evaporation under vacuum for 40 min (known as derivatization). The dried supernatant was then dissolved in 20 µL of ethanol/water/trimethylamine/phenylisothiocyanate solution (prior mixed at a ratio of 2:2:1) and immediately evaporated under vacuum for 40 min. Next, 30 µL of ethanol/water/triethylamine/phenylisothiocyanate solution (prior mixed at 7:1:1:1) were added into the sample and left for 20 min at room temperature to allow phenylisothiocyanate-GABA formation. The sample was vacuumed again for 40 min to remove excess solvent, then diluted and subjected to HPLC analysis. Mobile phase A (pH 5.8, adjusted using 0.1 M NaOH) consisted of 8.205 g of sodium acetate, 0.5 mL trimethylamine, and 0.7 mL acetic acid in 1 L of deionized water while mobile phase B consisted of 1 L of acetonitrile and deionized water mixed at a ratio of 3:2. Both mobile phases were filtered through a 0.45-μm membrane filter. Sample was injected at 5 µL and eluted at a flow rate of 0.6 mL/min using isocratic elution of 80% mobile phase A + 20% mobile phase B. Compound was identified at λ = 254 nm using a diode array detector. GABA content was calculated by comparing the sample peak area with that of GABA standard.

### 2.6. Determination of pH

All pH measurements were performed using a bench-top pH meter (model S20 SevenEasy^TM^, Mettler-Toledo GmbH, Columbus, OH, USA).

### 2.7. Determination of Water-Holding Capacity (WHC)

WHC was determined according to the modified procedure as described by Abdelmoneim et al. [20]. A sample of 10.0 g of yogurt (*W*_1_) was centrifuged at 5000× *g* for 10 min at 4 °C. The supernatant was collected and weighed (*W*_2_). WHC (%) was calculated as follows:

(1) $$\mathrm{WHC}\;=\;\frac{W_{1}-W_{2}}{\;W_{1}}\;\times 100\%.$$

### 2.8. Animal Study: Blood Pressure-Lowering Efficacy in SHR

Thirty-six male spontaneously hypertensive rats (aged 10 weeks old) were purchased from Animal Experimental Unit, Faculty of Medicine, University of Malaya (Kuala Lumpur, Malaysia) and acclimatized to laboratory conditions for 7 days prior to the experiment. All rats were housed in three per individually ventilated cage, at a room temperature of 22–24 °C, relative humidity of 50–60%, and maintained under an automated 12-h light/dark cycle. A standard fortified pellet diet (Altromin brand) and distilled water were available ad libitum.

The rats were randomly divided into 6 groups: positive control (captopril, 50 mg/kg, a pharmaceutical antihypertensive agent), negative control (distilled water), control yogurt (containing only starter culture, 30 mg/kg), and three doses of yogurt (30, 150, 300 mg/kg) corresponding to 0.1, 0.5, and 1.0 mg/kg GABA, respectively. The exact amount of yogurt fed to each rat was calculated based on their individual body weights. Weighed freeze-dried yogurt samples were dissolved in distilled water and topped up to 1.0 mL prior to administration using the oral gavage technique. Systolic blood pressure was measured after warming the animal at 37 °C for 10 min using a CODA non-invasive blood pressure system (Kent Scientific, CT, USA) by applying the tail-cuff method. Measurements were performed at 0 (before administration), 2, 4, 6, 8, and 24 h after administration. Rats were kept in a peaceful and calm condition prior to measurement. All procedures were performed according to the approval from Institutional Animal Care and Use Committee, Universiti Putra Malaysia (ethic no. UPM/IACUC/AUP-R072/2018).

### 2.9. Statistical Analysis

Analysis of variance (ANOVA) followed by Duncan’s test were performed to detect means at significant difference (*p* < 0.05) in all analyses. Student’s *t*-test was performed in RSM study to compare actual and predicted responses during model validation. Results were analyzed using Minitab^®^ statistical software version 16 (Minitab Inc., State College, PA, USA). All values were reported as mean ± standard deviation from at least triplicate determinations.

## 3. Results and Discussion

### 3.1. GABA-Producing Ability of Different LAB Strains

**Figure 1:**
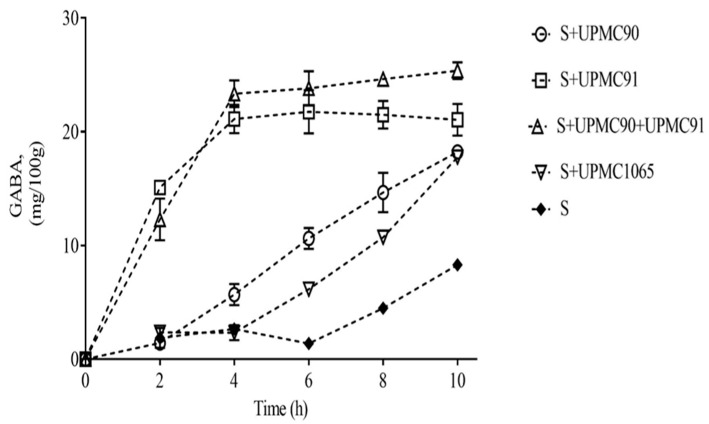
The changes in GABA content of five yogurt samples fermented at 5 mM glutamate and 36 °C for 10 h. S denotes starter culture containing only *S. thermophilus* and *L. delbrueckii* ssp. *Bulgaricus.*

Three different strains of *L. plantarum* Taj-Apis362 (two self-cloned UPMC90 and UPMC91, and one wild-type UPMC1065) were co-cultured with starter culture (a mixture of *S. thermophilus* and *L. delbrueckii* ssp. *bulgaricus*) to produce yogurt and evaluated the GABA-producing potential of these selected strains. Five yogurt samples were produced, namely control containing only the starter culture (S), S+UPMC90, S+UPMC91, S+UPMC90+UPMC91, and S+UPMC1065. Their respective GABA content and pH profiles, monitored over 10 h of fermentation, are shown in Figure 1. It is interesting to note that a combination of two self-cloned LAB strains (S+UPMC90+UPMC91) produced the highest GABA content (25.36 mg/100 g), followed by S+UPMC91 (21.05 mg/100 g), S+UPMC90 (18.23 mg/100 g), and S+UPMC1065 (17.76 mg/100 g) after 10 h of fermentation. In contrast, the control produced the lowest GABA content of 8.29 mg/100 g, which was significantly lower than all samples co-cultured with LAB strains. These findings revealed the different degree of GABA enhancement by all selected *L. plantarum* Taj-Apis362 strains in yogurt, indicating the potential of developing GABA-rich food through natural fermentation.

Meanwhile, all samples, with or without the presence of LAB, showed a concomitant reduction in pH, from pH 6.0 to 4.0, over 10 h of fermentation. This reduction is mostly contributed by the active metabolism of starter cultures, whereby *S. thermophilus* primarily dominates the early phase of fermentation at pH 6.5–5.5 and subsequently stimulates the growth of *L. delbrueckii* ssp. *bulgaricus* to reach a lower pH of 4.5–4.0. This acidic condition leads to the formation of gel particles in the yogurt, giving rise to a thick and curd-like consistency in the product [21,22]. The presence of LAB did not contribute significantly towards pH reduction in the samples. This is due to the fact that, even though lactic acid is constantly produced, the bacteria utilizes protons to support GAD enzyme activity for decarboxylation of glutamate into GABA, counteracting the effect of lactic acid and resulting in an unchanged pH [23,24]. For instance, S+UPMC90+UPMC91 produced the highest GABA content with pH similar to control (without the addition of LAB), suggesting that co-culturing LAB with high intracellular (UPMC90) and extracellular (UPMC91) GAD activity with starter culture significantly intensifies GABA production without jeopardizing the physicochemical quality. The increased conversion from glutamate to GABA during co-culturing pleasantly reveals the symbiotic relationship between starter culture and LAB to stimulate higher metabolic activity among the microorganisms [21]. This finding is in line with previous studies performed by Xiao et al. [25] and Hugenschmidt et al. [26], who used *Levilactobacillus brevis* strain NPS-QW 145 co-cultured with *Streptococcus thermophilus* to increase GABA production in milk and *Lactobacillus plantarum* SM39 co-cultured with *Propionibacterium freudenreichii* DF13 to produce high folate and vitamin B12, respectively. Taking this into account, S+UPMC90+UPMC91 was selected for further optimization study.

### 3.2. Optimization of Fermentation Parameters

Response surface methodology (RSM) was applied to optimize the fermentation parameters to produce GABA-rich yogurt. Three independent variables (temperature, glutamate substrate concentration, and incubation time) were chosen based on their dominant role to enhance GABA in GABA-producing microorganisms [27]. Three responses were monitored, i.e., GABA content, pH, and water-holding capacity (WHC). In bovine milk, 80% of protein is made up of casein micelles, of which their stability is highly dependent on the net negative charge and steric repulsion of κ–casein at natural milk pH [28]. During fermentation, the produced hydrogen ion (H^+^) neutralizes the negative charges, reducing the electrostatic repulsion between casein molecules to promote the formation of protein clusters and chains into a three-dimensional network, effectively entrapping water molecules to improve the WHC. Additionally, when pH reduces, the stabilizing layer surrounding casein molecules tends to collapse and weaken, bringing the molecules to a closer proximity, forming dense clusters and pact structures to facilitate gelling [29]. Thus, pH and WHC were selected as responses of interest in the current study due to their strong correlation with the gel-forming ability, which subsequently affects the yogurt quality.

The fermentation conditions (factors) and their corresponding actual and predicted GABA, pH, and WHC (responses) are detailed in Table 1. Statistical parameters, including *p*-value, *F*-value, lack-of-fit, and *R*-squared (*R*^2^), from multiple regression analysis were used complementarily to help identify the best-fitting model, of which the polynomial quadratic model was found to be the most suitable to describe the effect of temperature (*X*_1_), glutamate concentration (*X*_2_), and incubation time (*X*_3_) on the GABA, pH, and WHC of yogurt. From Table 2, the analysis of variance (ANOVA) revealed model *p*-value <0.0001 and *R*^2^ > 90% for all three responses, with *F*-values of 17.75, 431.32, and 45.65, for GABA, pH, and WHC, respectively. According to Karthikeyan et al. [30], a combination of a large *F*-value and small *p*-value suggested a significant effect on the respective response in any model. In addition, the *p*-values for lack-of-fit were insignificant (*p* > 0.05) for all measured responses. Adequate precision (measures the signal-to-noise ratio) of 16.24–64.80 was obtained in the current study and a ratio greater than 4 is considered as desirable to indicate adequate signal [31]. These observations suggest that the final reduced quadratic model sufficiently represented the obtained data and demonstrates high significance to predict any response accurately under any combination of fermentation parameters.

From Table 2, linear terms of temperature (*X*_1_) and incubation time (*X*_3_) had significant effects on GABA and pH (*p* < 0.05), while glutamate concentration (*X*_2_) had no effect on these responses (*p* > 0.05). WHC were significantly affected by *X*_2_ and *X*_3_ but not by *X*_1_. On the other hand, the quadratic effect of temperature (*X*_1_^2^) and glutamate concentration (*X*_2_^2^) was seen on all responses (*p* < 0.05), but that of time (*X*_3_^2^) was only observed on pH. As for interactive effects, *X*_1_*X*_3_ produced significant effect on all responses (*p* < 0.05), while *X*_1_*X*_2_ and *X*_2_*X*_3_ exerted a significant effect on GABA and pH, respectively. The final reduced model, containing only the significant terms, for GABA, pH, and WHC, is expressed as follows.

(2) $$Y_{1}=31.36+2.76X_{1}+1.14X_{2}+6.71X_{3}-6.08X_{1}^{2}-6.37X_{2}^{2}+2.53X_{1}X_{2}-2.96X_{1}X_{3},$$

(3) $$Y_{2}=3.95-0.14X_{1}+0.006X_{2}-0.37X_{3}+0.22X_{1}^{2}-0.11X_{2}^{2}+0.27X_{3}^{2}+0.11X_{1}X_{3}+0.03X_{2}X_{3},$$

(4) $$Y_{3}=64.49+0.84X_{1}+1.16X_{2}+2.93X_{3}-9.07X_{1}^{2}-2.42X_{2}^{2}-1.44X_{1}X_{3},$$

where responses are represented by *Y*_1_ = GABA (mg/100 g), *Y*_2_ = pH (unitless), and *Y*_3_ = WHC (%); and factors are represented by *X*_1_ = temperature (°C), *X*_2_ = glutamate concentration (mM), and *X*_3_ = incubation time (hour).

**Figure 2:**
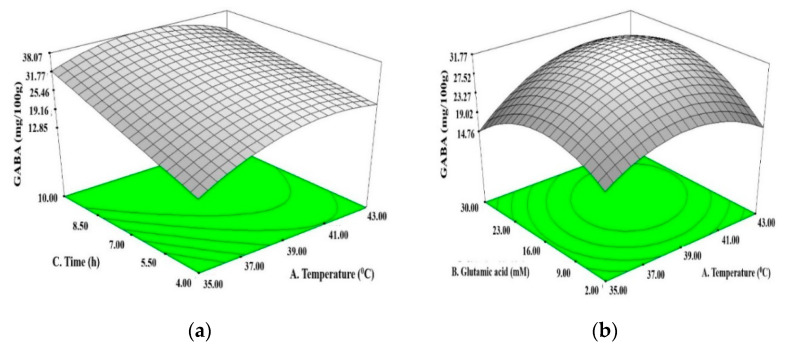

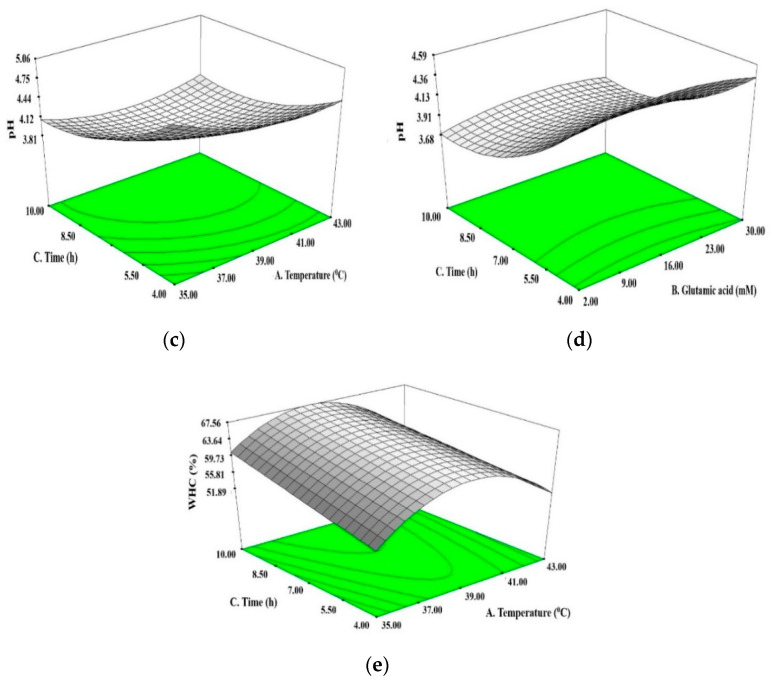
Three-dimensional response surface plots showing significant interaction between different factor combinations during fermentation; (**a**) Effect of temperature and glutamate on GABA, (**b**) Effect of temperature and time on GABA, (**c**) Effect of temperature and time on pH, (**d**) Effect of glutamate and time on pH; and (**e**) Effect of temperature and time on water-holding capacity.

All significant interactions on GABA, pH, and WHC, from different fermentation factor combinations, are illustrated as three-dimensional response surface plots in Figure 2. At a fixed incubation time of 7 h, the temperature–glutamate interaction effect positively enhanced GABA production, reaching a maximum at 39 °C and 16 mM glutamate, followed by a reduction when temperature and glutamate further increased to 43 °C and 30 mM, respectively (Figure 2a). These results implied that the interaction from temperature and glutamate played a critical role in enhancing GABA production in LAB strains, in line with that previously reported by Li et al. [32], who showed that temperature and glutamate significantly increased GABA production in *Lactobacillus* species. However, excessively high temperature and glutamate were unfavorable to an enhancement of GABA production, due to the restricted cell growth under extreme conditions [33,34]. Similarly, at a fixed glutamate concentration of 16 mM, the temperature–time interaction enhanced GABA production, which then reduced when the parameters further increased to 43 °C and 10 h (Figure 2b).

The contour plot in Figure 2c shows the temperature–time interaction effect on pH. At a fixed glutamate concentration of 16 mM, pH was inversely correlated to the temperature–time interaction, i.e., as temperature and time increased, the pH was lowered. This is due to the conversion of milk sugar (lactose) into lactic acid, as a result of bacterial metabolism during fermentation in the yogurt [35]. A similar inverse correlation was observed for the glutamate–time interaction effect on pH (Figure 2d), which recorded a reduced pH when glutamate and time increased. This may be attributed to the utilization of glutamate as a nitrogen source by bacterial cells to sustain growth, which produced lactic acid and lowered the pH as fermentation continued [36].

The contour plot in Figure 2e shows the temperature–time interaction effect on WHC, whereby WHC reached a maximum at 39 °C and 7 h but reduced gradually when the parameters further increased to 43 °C and 10 h. Kristo et al. [37] and Lazaridou et al. [38] observed that, at low fermentation temperatures, the hydrophobic interaction between casein micelles is weak, allowing protein molecules to gather in close proximity and form large clusters, building an extensive and strong protein network to produce a firm and dense gel structure that effectively immobilizes free water molecules in the gel matrix [39], thus improving the WHC. On the contrary, when the temperature continues to increase, hydrophobic interaction dominates, and casein micelles re further distanced apart, weakening the gel’s ability to entrap water molecules, and thus reducing the WHC. All selected parameters exerted significant linear, quadratic, and interaction effects on GABA, pH, and WHC, indicating that the fermentation parameters should be carefully manipulated to obtain GABA-rich yogurt with desirable product qualities.

### 3.3. RSM Model Validation

Model validation was performed following the predicted fermentation conditions to verify the accuracy and best-fit character of the selected model. The optimized conditions were predicted as follows: fermentation temperature = 39.0 °C, glutamate concentration = 11.5 mM, and incubation time = 7.25 h to yield GABA = 30.90 mg/100 g, pH = 3.90, and WHC = 64.11% theoretically. The actual experimental values obtained under these conditions were GABA = 29.96 mg/100 g, pH = 3.93, and WHC = 63.06%, respectively. According to Duttschaever et al. [40], normal pH values ranging from 3.27–4.53 have been reported for commercial yogurts and yogurts with WHC of 43.22–68.78% were found acceptable by experienced panelists [41]. The optimized pH and WHC obtained for GABA-rich yogurt were within the acceptable range. Student’s *t*-test revealed *p* > 0.05 for all comparisons, indicating no significant difference between actual and predicted values. This confirms the model’s significance and adequacy to predict the optimum fermentation parameters to produce GABA-rich yogurt with desirable pH and WHC.

### 3.4. Glucose Induction for Enhancing GABA Production

**Figure 3:**
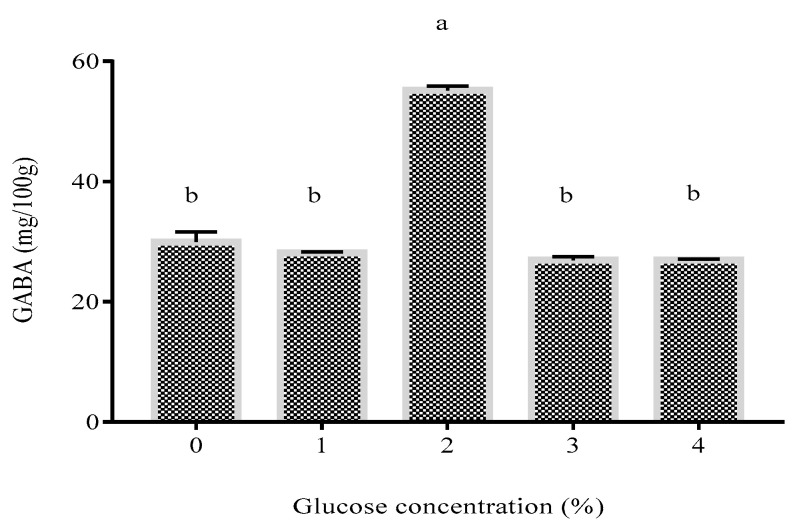
Effect of glucose induction at different concentrations on GABA content of yogurt. Different letters indicate a significant difference at *p* < 0.05.

Upon establishing the optimum fermentation conditions to produce GABA-rich yogurt, the effect of glucose to further enhance GABA production was investigated, whereby four levels of glucose (1–4%, *w*/*v*) were added into the yogurt prior to fermentation under optimized conditions. Figure 3 depicts that 2% glucose significantly induced GABA enhancement, in agreement with a previous study by Li et al. [42], who reported a glucose concentration of 2.5% as the best carbon level to enhance GABA production by *L. brevis* NCL912 in culture broth. In the present study, 2% glucose induced GABA production to 59.00 mg/100 g, a two-fold increment compared to that without glucose (29.96 mg/100 g). This finding is prominent and uplifting, as it strengthens the omission of expensive cofactor (pyridoxal-5-phosphate) from the fermentation medium and allows the minimal usage of glutamate substrate that would otherwise exert unfavorable savory/umami taste if present at a high concentration. In fact, the current work delightfully demonstrates the lowest glutamate concentration (11.5 mM) compared to previous work, which required up to 20–507 mM glutamate for GABA production [9,11,12]. In terms of incubation time, Chen et al. [43] reported a GABA yield of 7.30 g/L in fermented milk only after 72 h of incubation with *Streptococcus salivarius* subsp. *thermophiles fmb5* at 1.2% glutamate and 37 °C while Linares et al. [6] observed a GABA content of 2.20 mg/mL in yogurt cultured with *S. thermophilus* APC151 and *L. Bulgaricus* CH1 after 48 h of fermentation using 2.25 mg/mL glutamate at 42 °C. Meanwhile, Shan et al. [44] produced 231.23 mg/100 g of GABA in yogurt at a much shorter fermentation time of 5.75 h using commercial starter YC-X100 and *L. plantarum* NDC75017 at 36 °C, but it required a high concentration of glutamate (80 mM) and PLP (18 µM). In contrast to the lengthy fermentation time and usage of PLP as reported in previous work, the current study succeeded in reducing the fermentation time to 7.25 h without the use of PLP.

It is interesting to note that GABA production was not enhanced further when the glucose level increased to 3% and 4%, possibly due to the imbalanced osmotic pressure as a result of a high glucose concentration, which interfered with the bacterial metabolism, inhibiting growth and thus lowering the GABA-producing ability [45,46]. A similar study also showed increased GABA content with the addition of 1% glucose in fermented soymilk after 24 h [47]. While several studies have reported the effect of glucose in fermentation medium, they focused on the enhancement of fatty acid production from *Chlorella* sp. and biomass accumulation from green algae *Chlorella minutissima* [48,49]. To date, the effect of glucose to enhance GABA production in yogurt fermentation has not been reported elsewhere. Glucose is regarded as the optimal fermentable sugar for utilization as a substrate by LAB [50,51]. Therefore, the successful use of glucose to enhance GABA during LAB fermentation makes it an ideal choice to shorten the fermentation time, omit PLP, and allow minimal glutamate usage while favorably sustaining the growth of LAB without jeopardizing the product’s sensorial properties.

### 3.5. In Vivo Study: Blood Pressure-Lowering Efficacy in Rats

**Figure 4:**
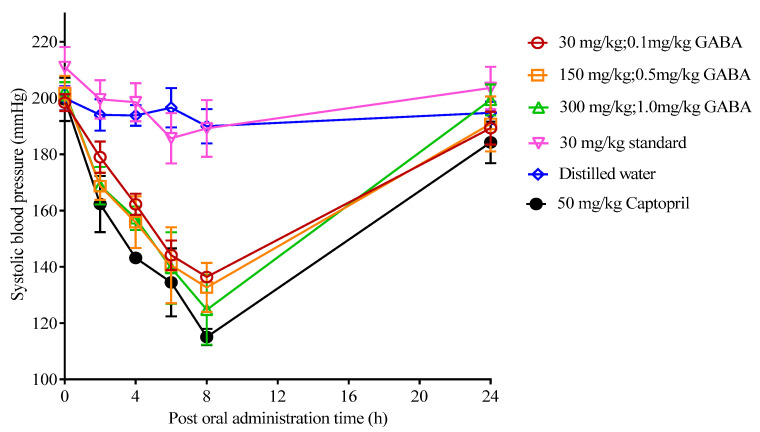
Mean systolic blood pressure of spontaneously hypertensive rats within 24 h of single oral administration of 30, 150, and 300 mg/kg yogurt (consisting of 0.1, 0.5, and 1.0 mg/kg GABA, respectively) compared to 30 mg/kg standard yogurt, 50 mg/kg captopril, and distilled water.

As GABA is known to reduce blood pressure in experimental animal and human studies, an animal study was performed to evaluate the blood pressure-lowering efficacy of GABA-rich yogurt in spontaneously hypertensive rats (SHRs). Systolic blood pressure (SBP) was measured within 24 h following single oral administration of yogurts. A total of six samples were evaluated, namely GABA-rich yogurt at three different concentrations of 30, 150, and 300 mg/kg (corresponding to 0.1, 0.5, and 1.0 mg/kg GABA, respectively), standard yogurt comprising only the starter culture (for yogurt-basis comparison), captopril (50 mg/kg, positive control), and distilled water (negative control). From Figure 4, the captopril group depicted the most prominent SBP-lowering effect, in line with its function as an antihypertensive agent while the distilled water group depicted no changes in SBP throughout the monitoring period. All three GABA dosages synchronously revealed the highest SBP reduction at 8 h post administration, recording values of −65.22, −66.09, and −77.59 mmHg for 0.1, 0.5, and 1.0 mg/kg GABA, respectively. It is also noted that the SBP in all GABA groups returned to baseline after 24 h, indicating that GABA was fully degraded upon gastrointestinal digestion without a cumulative effect in the body.

From Table 3, the blood pressure-lowering efficacies between three GABA dosages depicted no significant differences (*p* > 0.05) at all measurement hours, establishing the effective GABA concentration at 0.1 mg/kg (equivalent to 30 mg/kg GABA-rich yogurt) to exert a blood pressure-lowering effect in SHR. This dosage is lower than that reported by Hayakawa et al. [5] and Yamakoshi et al. [52], who fed SHR with a single oral dosage of 0.05–5.00 mg/kg and 0.33–3.30 mg/kg GABA from fermented milk and soy sauce, respectively, to exert blood pressure-lowering activity. The blood pressure-lowering effect may be contributed by the reduction of oxidative stress in the rats. Previously, Kawakami et al. [53] reported the decrease in plasma 8-hydroxydeoxyguanosine and urinary isoprostane levels, two important compounds synthesized as a result of excessive oxidative stress [54], and act as markers for damaged oxidative cell membrane phospholipid [55], in SHRs fed with GABA-enriched brown rice. This indicated the positive correlation between consumption of GABA-rich food and reduction of oxidative stress.

Yogurt, as a protein-rich food, contains various peptides generated from bacterial metabolism, among which some may possess biological activity to exert blood pressure-lowering activity. Thus, standard yogurt was fed to SHR to evaluate the antihypertensive effect, if any, from the pool of peptide mixture generated during fermentation. The SBP readings in standard yogurt showed no significant difference from distilled water, indicating no antihypertensive activity in the yogurt sample. This observation confirms that the blood pressure-lowering activity in GABA-rich yogurt was due to the presence of GABA, irrespective of peptides, fostering the potential of GABA-rich yogurt as a functional dairy product that can be consumed regularly to promote blood pressure-lowering functionality.

## 4. Conclusions

Co-culturing starter culture with GABA-producing LAB strains (UPMC90 + UPMC91) successfully established a symbiotic relationship between these species that intensified GABA production in yogurt. Glucose induction at 2% (*w*/*v*) under optimized fermentation conditions delightfully produced a yogurt with increased GABA content and desirable product quality (pH and water-holding capacity). The lowered blood pressure in SHR upon single oral administration of GABA-rich yogurt signified its potential to be developed into a functional dairy product possessing a valuable antihypertensive property.

## Figures and Tables

**Table 1:** Central composite design with actual experimental and predicted response values for yogurt fermented by S+UPMC90+UPMC91 LAB strains.

Run	Factor			Response					
	*X*_1_, (°C)	*X*_2_, (mM)	*X*_3_, (hour)	*X*_3_, (hour)	Actual GABA (mg/100g)	Predicted GABA (mg/100 g)	Actual pH	Predicted pH	Actual WHC (%)	Predicted WHC (%)
1	39	30	7	29.03	26.13	3.81	3.84	63.87	63.24
2	39	16	4	25.03	24.65	4.55	4.59	61.41	61.57
3	39	16	7	31.25	31.36	3.96	3.95	63.47	64.49
4	39	16	7	30.98	31.36	3.92	3.95	64.91	64.49
5	35	30	4	0.00	5.10	4.93	4.92	50.25	50.64
6	39	16	7	31.12	31.36	3.95	3.95	63.82	64.49
7	39	16	7	32.53	31.36	3.97	3.95	62.37	64.49
8	35	30	10	27.28	24.43	3.99	4.01	57.54	59.38
9	35	2	4	10.00	7.87	4.98	4.97	47.69	48.32
10	35	2	10	24.32	27.20	3.94	3.93	57.17	57.06
11	39	16	10	32.57	38.07	3.85	3.84	68.67	67.42
12	43	2	4	12.85	14.26	4.47	4.47	48.43	49.52
13	43	30	10	28.41	29.10	3.98	3.96	54.08	54.82
14	39	16	7	29.31	31.36	3.96	3.95	65.26	64.49
15	43	2	10	24.02	21.76	3.87	3.89	53.78	52.49
16	35	16	7	25.52	22.52	4.30	4.30	59.02	56.27
17	39	16	7	35.32	31.36	3.98	3.95	65.09	64.49
18	43	16	7	27.84	28.05	4.00	4.03	52.78	54.59
19	43	30	4	21.63	21.59	4.44	4.42	54.20	51.85
20	39	2	7	23.74	23.85	3.83	3.83	61.23	60.91

*X*_1_: temperature; *X*_2_: glutamate concentration; *X*_3_: time.

**Table 2:** Analysis of variance (ANOVA) for the full quadratic model of GABA content, pH, and water-holding capacity obtained during optimization.

	GABA		pH		WHC	
	*F*-Value	*p*-Value	*F*-Value	*p*-Value	*F*-Value	*p*-Value
Model	17.75	<0.0001	431.32	<0.0001	45.65	<0.0001
*X*_1_	7.41	0.0185	275.24	<0.0001	2.78	0.1192
*X*_2_	1.26	0.2832	0.52	0.4858	5.37	0.0375
*X*_3_	43.68	<0.0001	2021.62	<0.0001	33.75	<0.0001
*X*_1_^2^	11.47	0.0054	189.20	<0.0001	103.86	<0.0001
*X*_2_^2^ *X*_3_^2^	12.60	0.0040	49.70 285.86	<0.0001 <0.0001	7.32	0.0180
*X*_1_ *X*_2_	4.95	0.0459				
*X*_1_ *X*_3_ *X*_2_ *X*_3_	6.78	0.0230	152.91 10.41	<0.0001 0.0081	6.56	0.0237
Lack of fit	3.59	0.0891	2.14	0.2107	2.58	0.1558
*R*_2_	91.19%		99.68%		95.47%	
*R*_2_ adjusted	86.06%		99.45%		93.38%	

Note: *X*_1_ = Temperature (°C), *X*_2_ = Glutamate concentration (mM), *X*_3_ = Time (hour).

**Table 3:** Difference in systolic blood pressure (mmHg) in spontaneously hypertensive rats after single oral administration of yogurt.

Time (h)	Distilled Water	Captopril	Standard Yogurt	GABA Dosage (mg/kg)		
				0.1	0.5	1.0
2	5.46 ± 8.69 ^b^	37.22 ± 10.00 ^a^	21.60 ± 6.58 ^ab^	19.37 ± 6.69 ^ab^	30.76 ± 14.17 ^a^	33.50 ± 14.76 ^a^
4	6.21 ± 5.74 ^d^	56.38 ± 6.67 ^a^	20.02 ±13.18 ^cd^	36.10 ± 5.89 ^bc^	43.59 ± 3.12 ^ab^	45.07 ± 11.19 ^ab^
6	3.68 ± 2.76 ^b^	69.81 ± 10.82 ^a^	26.79 ± 8.88 ^b^	60.35 ± 7.46 ^a^	9.28 ± 17.99 ^a^	62.77 ± 16.87 ^a^
8	7.77 ± 2.41 ^c^	84.49 ± 9.99 ^a^	24.57 ± 3.42 ^c^	5.22 ± 8.31 ^b^	66.09 ± 8.40 ^b^	77.59 ± 15.89 ^ab^
24	4.54 ± 0.72 ^a^	15.32 ± 11.03 ^a^	6.16 ± 8.20 ^a^	12.33 ± 3.37 ^a^	11.55 ± 8.24 ^a^	1.80 ± 0.28 ^a^

The values are mean differences± standard deviations of systolic blood pressure (mmHg) from baseline reading (0 h) of 30, 150, and 300 mg/kg yogurt (consisting 0.1, 0.5, and 1.0 mg/kg GABA, respectively) in comparison with 30 mg/kg standard yogurt, 50 mg/kg captopril, and distilled water. Different letters within the same row indicate significant differences (*p* < 0.05) among samples at the same monitoring hour.
